# Immunotherapy in Testicular Germ Cell Tumors

**DOI:** 10.3389/fonc.2020.573977

**Published:** 2020-09-24

**Authors:** Katarina Kalavska, Silvia Schmidtova, Michal Chovanec, Michal Mego

**Affiliations:** ^1^Translational Research Unit, Faculty of Medicine, Comenius University and National Cancer Institute, Bratislava, Slovakia; ^2^Department of Molecular Oncology, Cancer Research Institute, Biomedical Research Center, University Science Park for Biomedicine, Slovak Academy of Sciences, Bratislava, Slovakia; ^3^Second Department of Oncology, Faculty of Medicine, Comenius University and National Cancer Institute, Bratislava, Slovakia

**Keywords:** testicular germ cell tumors, cisplatin-resistance, immunotherapy, immune checkpoint inhibitors, immune microenvironment

## Abstract

Testicular germ cell tumors (TGCTs) are malignancies with very high curative potential even in metastatic settings, mainly due to the introduction of cisplatin in the treatment of this disease. However, in a group of patients with cisplatin-refractory disease or with progressive disease despite high-dose salvage chemotherapy treatment, the prognosis is typically dismal. The triple combination of gemcitabine, oxaliplatin, and paclitaxel (GOP) has reasonable efficacy and is considered to be standard care for this group of patients. It remains to be seen, however, whether refractory TGCTs may represent a potential target for immune checkpoint inhibition. This review will focus on the rationale of the use of immunotherapy for platinum-refractory TGCTs and summarize data reporting experiences with immune checkpoint inhibitor treatment for this malignancy.

## Introduction

Testicular germ cell tumors (TGCTs) are considered the most frequent solid malignancy among young men aged 34 years (1). These tumors represent an excellent example of a cancer disease with high curative potential, particularly due to their high sensitivity to cisplatin-based chemotherapy (2).

Treatment for TGCTs is selected with regards to the tumor subtype and stage. While orchiectomy followed by surveillance is an adequate and predominant treatment option in these patients, metastatic TGCTs are treated by chemotherapy alone or a combination of chemotherapy, surgery, and in a few cases, radiation therapy (3). Approximately 15–20% of patients with metastatic disease relapse after initial chemotherapy. However, ~50% of this patient group can achieve cure by salvage treatment (3, 4). Last, there is a group of patients with cisplatin-refractory disease or who relapse after second-line chemotherapy. For these patients, the prognosis of the disease is poor, and they are usually treated with novel chemotherapy regimens (5, 6). However, despite the existence of numerous new treatment options involving targeted and biological therapies (sorafenib, pazopanib, everolimus, sirolimus/erlotinib, and brentuximab) that have been evaluated in cisplatin-refractory TGCTs, conventional chemotherapy options with limited activity continue to be utilized for these patients (7).

Several immune checkpoint inhibitors have already been assessed in various types of cancers, including advanced urothelial cancer, metastatic renal cell carcinoma, and genitourinary cancers (8–13).

This review will focus on the rationale of the use of immunotherapy for platinum-refractory TGCTs and summarize data evaluating the use of immune checkpoint inhibitors in the treatment of these patients.

## Rationale for Immunotherapy in Testicular Cancer

The mammalian testes are characterized as immunologically privileged sites, mainly due their special immunological environment that protects germ cells from autoimmune attack and a deficiency in the response of the testicular immune system to antigens. Moreover, the mechanisms underlying the immune privilege of the testis seem to also be involved in the control of spermatogenesis and steroidogenesis (14). Interestingly, a study published by Dorantes-Heredia et al. (15) describes a rare case of burned-out testicular tumor in a 34-year-old patient. Histological analysis of primary tumor tissue isolated *via* unilateral orchiectomy for this patient showed no pathological alteration within the examined tissue, while a heterogeneous retroperitoneal metastatic mass persisted. The phenomenon of spontaneous testicular tumor regression without any treatment is thought to be linked to the host's immune microenvironment as well as to amended vascularization of the tumor (15). In addition, data presented by Pearce et al. indicate that testicular cancer patients develop specific immunological responses against cancer/testis antigens (CTAg), predominantly mediated by strong CD8+ and CD4+ CTAg-specific T-cell responses. These effector T cells are poorly detectable in the absence of CTAg, and their level decreases substantially after treatment (16). However, a possible link between immune privilege and the development of testicular germ cell tumors remains unrecognized (17).

The specific immune reaction to the presence of germ cell neoplasia *in situ* (GCNIS) and overt germ cell tumor (GCT) has been assessed *via* characterization of immune cells as well as cytokine profiles within the tissue in several works. Klein et al. described a significantly different pattern of immune cell distribution in testicular germ cell tumors compared to normal testis or inflammatory lesions related to hypospermatogenesis. While T cells were detected in all analyzed samples, the immune microenvironment of testicular tumors was uniquely characterized by the presence of B cells and dendritic cells. Moreover, a cytokine profile characterized by increased transcript levels of IL-6 and other B cell supporting or T-helper cell type 1 (Th-1)-driven cytokines was described in only GCNIS and seminoma samples but not in normal spermatogenesis and hypospermatogenesis samples. Particularly, high levels of transcripts encoding pro-inflammatory cytokines (IL-1β, IL-6, and TNF-α), anti-inflammatory cytokines (TGF-β1), Th1-driven cytokines (IL-2 and IFN-γ), and chemokines (CXCL-13, CXCL-10, and CCL-5) were detected only in malignant cases (18). Contrary to these results, data published by Hvarness et al. support the absence of active immune surveillance in TGCTs. They showed a similar composition of infiltrating immune cells, involving the presence of macrophages, CD8+ and CD45R0+ T lymphocytes and B-lymphocytes in testicular tissue isolated from infertile men without neoplasia and GCNIS (19). Interestingly, a study published by Cheng et al. demonstrated that programmed death-ligand-1 (PD-L1) is constitutively expressed in the testis and, *via* programmed death-1 receptor (PD-1)/PD-L1 negative costimulation, mediates immune privilege and prolongs the survival of intratesticular islet allografts (20). Opposite results were obtained by Fankhauser et al., who examined PD-L1 expression first in germ cell tumors. PD-L1 expression was not detected in any of the normal tissue specimens or precursor GCNIS lesions. However, 73% of all evaluated seminomas and 64% of nonseminomas exhibited abundant expression of PD-L1 (21). Furthermore, tissue microarray-based analysis enrolling 84 patients also demonstrated significant upregulation of PD-L1 expression as well as infiltration of PD-1-positive cells in testicular tumors with respect to that of normal-appearing testis tissue. The results of this study also indicated that PD-1-positive cytotoxic cells may require pathologic tumor vessels to migrate through the blood-testis barrier into the tumor (22).

Similar results reporting increased PD-L1 expression in TGCTs were achieved by our group as well. Namely, this work showed significantly higher expression of PD-L1, but not PD-1, in TGCTs than in normal testicular tissue. However, it is important to note that inconsistent scoring systems and various antibodies have been used in individual studies. Moreover, our findings reporting the prognostic significance of the PD-1/PD-L1 signaling pathway highlighted the importance of PD-L1 in the immune tolerance of germ cell tumors (GCTs) and the facilitation of tumor dissemination. The abundant expression of PD-L1 in primary tumor tissue correlated with poor-risk clinical characteristics defined by International Germ Cell Cancer Collaborative Group (IGCCCG) classification, including ≥3 metastatic sites, increased serum tumor markers, and the presence of nonpulmonary visceral metastases. Conversely, patients with low PD-L1 expression showed a better outcome, namely, better progression-free survival and overall survival (23). In addition, a study investigating PD-1 and PD-L1 expression on tumor infiltrating lymphocytes (TILs) and their prognostic role in TGCTs revealed that primary testicular TGCT patients with increased expression of PD-L1 on TILs had significantly better outcomes than patients with lower expression. The hypothesis that immune escape of testicular cancer cells followed by disease dissemination is a result of lower PD-L1 expression on TILs was also supported by a significant correlation between lower PD-L1 expression on TILs and poor-risk disease according to the IGCCCG (24). The association between inflammatory markers and tumor stage was also evaluated in a study by Imamoglu et al., which included 66 seminomatous and 46 nonseminomatous GCT patients undergoing inguinal orchiectomy. Statistically significant differences were shown between the median neutrophil-to-lymphocyte ratio (NLR), lymphocyte-to-monocyte ratio (LMR), and systemic immune-inflammation index (SII) in early vs. advanced stages of seminomatous tumors, while within the nonseminomas, only a difference between the median platelet-to-lymphocyte ratio (PLR) was described. These data are in concordance with findings describing more intense immune cell infiltration in seminomatous than in nonseminomatous testicular tumors (25). Furthermore, it was also established that leukocyte and platelet counts might predict a poor outcome in TGCT patients. In particular, an increased NLR and elevated absolute platelet and absolute neutrophil counts were associated with advanced stages of disease (stage II–III). Absolute neutrophil counts also correlated with more frequent progression of disease and mortality (26). Interestingly, Chovanec et al. performed a combinatorial survival analysis involving the systemic immune-inflammation index (SII) based on platelet, neutrophil and lymphocyte counts and PD-L1 expression on tumor infiltrating lymphocytes (TILs). This analysis was able to recognize three distinctive prognostic groups (never relapsing, never dying, and poor prognostic groups). A favorable prognosis was possibly mediated by PD-L1 expression on TILs, which could reduce the proinflammatory environment, and vice versa. Therefore, we cannot determine whether systemic inflammation forms a permissive microenvironment resulting in the manifestation of disease with poor prognostic features or if the SII is a consequence of aggressive disease (27).

Characterization of the immune infiltrate in terms of immune checkpoint PD-L1/CTLA-4 expression and its correlation with patient outcome was carried out by Lobo et al. A predominant portion of 162 analyzed samples exhibited PD-L1 and CTLA-4 positivity in infiltrating immune cells (ICs) regardless of the histological subtype, while the expression of CTLA-4 in tumor cells (TCs) was significantly higher in yolk sac tumor, choriocarcinoma, and teratoma samples. The most frequent PD-L1 TC positivity was determined in choriocarcinomas (28). These results are in line with data from Sadigh et al., where choriocarcinoma was the only GCT histological subtype expressing PD-L1 in tumor cells, whereas other subtypes primarily expressed varying levels of PD-L1 on tumor-associated macrophages (TAMs) without true PD-L1 expression on tumor cells themselves. Moreover, this study also revealed a significantly higher expression of PD-L1 on TAMs in seminomatous than in nonseminomatous samples (29). The absence of PD-L1 positivity in ICs was also determined to be an independent predictor of worse relapse-free survival (RFS) when adjusting for several other clinical variables. In addition, a similar pattern was also determined for CTLA-4 immunoexpression in ICs. Namely, higher CTLA-4 expression correlated with good prognostic features, including lymphovascular invasion and lower pT and N stages (28). Moreover, Yamada et al. showed the prognostic value of CD66b + tumor-infiltrating neutrophils (TINs) in 102 patients who underwent orchiectomy due to testicular cancer. Additionally, a positive association between increased CD66b + TIN density and the presence of metastasis, S stage, and nonseminomatous histology was determined in this analysis (30).

The importance of the PD-1/PD-L1 signaling pathway in immune escape of testicular cancer was also explored by Siska et al. Multiplexed fluorescence immunohistochemistry (FIHC) for T-cell subsets and immune checkpoints as well as targeted gene expression profiling were used for the deep characterization of the immune infiltrate. Activated CD3+ T-cell infiltration, increased PD-L1 expression, and elevated PD-1/PD-L1 spatial interaction were predominantly found in seminomas and correlated with a good prognosis of the disease. On the other hand, high neutrophil, and macrophage gene signatures were described in nonseminomas. Regardless of the histological tumor subtype, advanced stages of disease were characterized by decreased T-cell and NK-cell signatures, while Treg, neutrophil, mast cell, and macrophage signatures were elevated in these cases (31). The aforementioned data shed more light on the use of immune checkpoint inhibition as a novel treatment option for patients with refractory TGCTs.

The overview of studies determining PD-1/PD-L1 expression on tumor cells and/or immune cells with additional information about the used antibodies/assays is shown in Table 1.

**Table 1:** Overview of studies determining PD-1/PD-L1 expression on tumor cells and/or immune cells with additional information about the used antibodies/assays.

**Reference**	**PD-1/PD-L1 expression and its correlation to patient's outcome (when available)**	**Antibody/assay**
(20)	- PD-L1 constitutively expressed in testis	Immunohistochemistry using the goat anti-mouse PD-L1 antibody (R&D Systems) Real-time polymerase chain reaction
(21)	- PD-L1 expressed in none of normal tissue specimens or GCNIS PD-L1 expressed in 73% of evaluated seminomas and 64% non-seminomas	Immunohistochemistry using the monoclonal rabbit antibody (E1L3N, Cell Signaling Technology, Inc. (CST), Danvers, MA, USA)
(22)	- Significant upregulation of PD-L1 expression in testicular tumors compared with normal appearing tissue	Immunohistochemistry with the primary antibody [PD-L1: Cell Signaling rabbit monoclonal antibody (no. 13684; Cell Signaling Technology, Inc., Leiden, Netherlands); PD-1: Abcam mouse monoclonal antibody (no. ab52587; Abcam Cambridge, UK)].
(23)	- Correlation of increased PD-L1, but not PD-1, expression in TGCTs with poor clinical characteristics	Immunohistochemistry with the primary mouse monoclonal antibody against PD-1 [Abcam (NAT105): AB52587] and rabbit monoclonal antibody against PD-L1 (Abcam [EPR1161(2)]: AB174838)
(24)	- Lower PD-L1 expression on TILs correlated with poor risk disease	Immunohistochemistry with the primary mouse monoclonal antibody against PD-1 (Abcam (NAT105): AB52587) and rabbit monoclonal antibody against PD-L1 (Abcam [EPR1161(2)]: AB174838)
(28)	- PD-L1/CTLA-4 positivity in ICs regardless the histological subtype (independent predictor of worse RFS) - PD-L1 expression in TCs—only in choriocarcinomas	Immunostaining with the anti PD-L1 antibody (Dako, clone 22C3)
(29)	- PD-L1 expression in TCs—only in choriocarcinomas - Higher PD-L1 expression in TAMs in seminomas when compared to non-seminomas	Immunostaining with the anti PD-L1 (Cell Signaling (E1J2J): 15165BF) and anti PD-1 Abcam (NAT105): AB52587 antibody
(31)	- Increased PD-L1 expression and elevated PD-1/PD-L1 spatial interaction predominantly found in seminomas and its correlation with good prognosis disease	Multiplexed fluorescence immunohistochemistry (FIHC) combined with automated quantitative analysis (AQUA; Genoptix, Inc.) Targeted gene expression profiling (Nanostring nCounter Immune panel)

## The Use of Immune Checkpoint Inhibitors in the Treatment of TGCTs

PD-1 belongs to the family of T-cell regulators and is expressed on double-negative T cells in the thymus and on activated CD4+ T cells, CD8+ T cells, natural killer cells, B cells, and monocytes (32). PD-L1 is a PD-1 ligand involved in the modulation and maintenance of the balance between T-cell activation and immune tolerance *via* interaction with the PD-1 receptor (33). PD-L1 represents an important mechanism by which cancer cells are able to suppress antitumor immunity in the tumor microenvironment; therefore, alterations in the PD-1 signaling pathway have a great impact on immunological homeostasis (34). Inhibition of the cytotoxic T-cell response is mediated *via* the interaction between PD-L1 or PD-L2 expressed on tumor cells and the PD-1 receptor on T-cells (33). The mechanism of action of checkpoint inhibition is based on the blockade of inhibitory signals, mainly due to the inhibition of the link between PD-1 and PD-L1 or PD-L2. This blockade results in a nonspecific reactivation of T-cells and tumor-specific T-cells, which are again able to attack tumors (35).

### Anti-PD-1/PD-L1 Inhibitors in TGCTs

The possible use of immunotherapy in metastatic germ cell tumors was described in a number of case reports and case series. The efficacy of immune checkpoint inhibitors as reported in available case reports and clinical trials is summarized in Table 2. These data are reviewed in the work of Semaan et al. (7, 44). Briefly, Shah et al. reported the clinical response of patients with embryonal cell carcinoma following a single dose of anti-PD-1 immunotherapy. The authors described 33% tumor regression in tumor volume based on RECIST version 1.1 and 49% reduction by immune-related response criteria (36).

**Table 2:** Efficacy of immune checkpoint inhibitors evaluated in single case reports, case report series, and clinical trials.

**Treatment**	**Target**	**No. of patients**	**Type of study**	**Response to the treatmentN (%)**	**Author (reference)**	**Comment**
Nivolumab	Anti-PD-1	1	Case report	1 (100%)	(36)	Single dose of nivolumab
PembrolizumabNivolumab	Anti-PD-1	7	Case report series	3 (43%)	(37)	1 pt—partial radiographic response1 pt—stable disease1 pt—mixed response
Nivolumab	Anti-PD-1	1	Case report	1 (100%)	(38)	Partial radiographic response in lung mts
Pembrolizumab	Anti-PD-1	1	Case report	0 (0%)	(39)	Rapid progression of disease
Brentiximab-vedotin + Pembrolizumab	Anti CD30 Anti-PD-1	1	Case report	1 (100%)	(40)	Partial remission (therapy terminated due to toxicity)
Pembrolizumab	Anti-PD-1	12	Clinical trial	0 (0%)	(41)	Trial terminated due to inefficacy
Durvalumab vs. Durvalumab + Tremelimumab	Anti-PD-L1 Anti-PD-L1 + Anti-CTLA-4	11 (arm A)11 (arm B)	Clinical trial	0 (0%)2 (18%)	(42)	100% progression in arm A
Avelumab	Anti-PD-L1	8	Clinical trial	0 (0%)	(43)	Trial terminated due to inefficacy

A large case series evaluating immunotherapy in refractory TGCTs presented by Zschäbitz et al. included seven platinum-refractory germ cell cancer patients who relapsed after high-dose chemotherapy and stem cell transplantation. Patients were treated with nivolumab or pembrolizumab. Four patients died shortly after single-dose treatment due to tumor progression. Partial radiographic response was achieved in one of the three remaining patients. However, it is important to consider that concomitant etoposide therapy was administered to this patient (37).

A durable response to the immune checkpoint inhibitor nivolumab in a man with poor-risk metastatic choriocarcinoma was presented in a case report by Chi et al. The patient documented in this case report received multiple lines of chemotherapy and radiotherapy, including four cycles of VIP (etoposide, ifosfamide, and cisplatin), stereotactic radiosurgery and high-dose chemotherapy with autologous stem cell transplantation. Nivolumab was administered over 14 months. After treatment, persistent partial radiographic disease stability and constant levels of b-HCG were described (38).

In another case, the authors reported the use of pembrolizumab in a patient with cisplatin refractory metastatic choriocarcinoma. Due to relapse after three different lines of standard chemotherapy, he was included in a phase II clinical trial and received 200 mg IV pembrolizumab. However, three weeks after the first pembrolizumab administration, his disease progressed rapidly, and immunotherapy with pembrolizumab was terminated (39).

Adra et al. performed the first single-arm phase II study investigating the anti-PD1 antibody pembrolizumab. This study involved 12 patients with incurable, cisplatin-refractory nonseminomatous germ cell tumors irrespective of their PD-L1 expression. Only 2 of 12 enrolled patients were characterized as having PD-L1-positive tumors. Although two patients (excluding those who were PD-L1 positive) achieved a mixed response with radiographic stability for 28 and 19 weeks, no partial or complete remission was observed using this treatment among the recruited patients (41).

In another open label, phase II clinical trial, the anti-PD-L1 inhibitor durvalumab, alone or in combination with tremelimumab (anti-CTLA-4 inhibitor), was evaluated. In total, 22 patients were recruited into this study (11 in arm A with durvalumab alone and 11 in arm B with a combination of durvalumab plus tremelimumab). Because of the significant number of patients (72.7%) exhibiting hyperprogressive disease under monotherapy with durvalumab, monotherapy arm A was closed to accrual. One patient in arm B achieved partial response in multiple lung metastases. Another patient in this arm had stable disease with serum tumor marker decline. All the remaining patients experienced progression of the primary tumor, while hyperprogressive disease was described in four (36.4%) of them. In addition, response and progression occurred regardless of tumor molecular features and PD-L1 expression (45).

Finally, a phase II study of avelumab (anti-PD-L1 inhibitor) was performed in patients with multiple relapsed and/or refractory germ cell tumors. The primary endpoint was 12-week progression-free survival. Eight heavily pretreated patients predominantly with nonseminomatous germ cell tumors were enrolled in this study. However, the primary endpoint (12-week PFS) could not be achieved in this prospective phase II clinical trial, and no subjective response or disease stabilization was observed in the analyzed cohort of patients (43). Immunotherapy using the anti-PD-L1 inhibitor avelumab was also evaluated in 15 patients with gestational trophoblastic tumors (GTTs) resistant to monochemotherapy with methotrexate or actinomycin-D. Treatment with avelumab was effective, and a favorable safety profile with respect to chemotherapy was observed in all enrolled patients. According to these data, approximately 50% of patients with chemoresistant GTT in this study could be cured of their chemoresistant diseases by immunotherapy (46).

Several active clinical trials using immune checkpoint inhibitors in cancer patients are currently underway; the overview of trials enrolling GCT patients who failed chemotherapy is summarized in Table 3.

**Table 3:** Overview of ongoing clinical trials evaluating immunotherapy use in cancer patients, including GCT patients.

**NCT number**	**Target**	**Drug**	**Phase**	**Condition/disease**	**Estimated study completion date**
NCT02832167	Anti-PD-1	Nivolumab	Phase II	Basket trial (various tumor types)	June 30, 2020
NCT02496208	Anti-PD-1 cMET Anti-CLA-4	Nivolumab plus and Cabozantinib with or without Ipilimumab	Phase I	Basket trial (genitourinary tumors)	September 30, 2020
NCT02834013	Anti-CLA-4 Anti-PD-1	Ipilimumab plus and Nivolumab	Phase II	Basket trial (rare tumors)	August 31, 2021
NCT03158064	Anti-PD-L1 Anti-CLA-4	Durvalumab and Tremelimumab	Phase II	Relapsed or refractory germ cell tumors	November 2021
NCT03333616	Anti-PD-1 Anti-CLA-4	Nivolumab plus and Ipilimumab	Phase II	Basket trial (rare genitourinary malignancies)	May 31, 2025

### Immunotherapy of TGCTs Beyond PD-1/L1 and CTL-4 Inhibition

#### Conjugated Antibodies

The antitumor effect of brentuximab-vedotin (BV) (anti-CD-30 antibody-drug conjugate comprising a chimeric antibody bound to cell-surface antigen CD30 covalently conjugated to the cytotoxic antitubulin agent monomethyl auristatin E) was evaluated in a case series published by Albany et al. Seven patients with relapsed or refractory CD30-expressing germ cell tumors or metastatic sex cord stromal tumors received brentuximab-vedotin at starting doses of 1.8 or 2.4 mg/kg every 3 weeks within a phase II study (NCT01461538). In two of seven enrolled patients, objective response was reported. One patient achieved complete response after four cycles of therapy with brentuximab-vedotin persisting for more than 46 months after disruption of treatment. In one patient, partial response to the therapy following two cycles of treatment was reported. However, after four cycles of brentuximab-vedotin, the therapy was discontinued because of the detection of progressive disease (47).

Furthermore, the antitumor effect of brentuximab-vedotin was also assessed in another phase II trial enrolling 24 patients with CD30+ germ cell tumors. At the end of the first stage of the reported study, nine patients had been treated with BV (three in the third line, six beyond the third line). A decrease in serum tumor markers was detected in seven patients after the first dose and in four patients after two doses. The 3-month progression-free survival was 11.1%, and the 6-month overall survival was 85.7% (48).

Nevertheless, it is important to note that most of the reported responses recorded after the use of the abovementioned immune checkpoint inhibitors (anti-PD-1/PDL-1 and anti-CTL-4) as well as the CD30-targeted inhibitor BV tended to be short-lived.

Data presented in the case report by Mayrhofer et al. are the first to evaluate a combination treatment with brentuximab-vedotin (and pembrolizumab, an immunotherapeutic agent) in a patient with metastatic extragonadal embryonal carcinoma. The patient was treated with three cycles of chemotherapy with BEP (bleomycin, etoposide, and cisplatin). Subsequently, due to duodenal metastasis recurrence with retroperitoneal lymphadenopathy, the patient underwent treatment with two cycles of ifosfamid/paclitaxel and carboplatin/etoposide with autologous stem cell support, which led to complete remission confirmed by CT scans. Five months later, two liver metastases were detected. The patient received chemotherapy with gemcitabine, oxaliplatin, and docetaxel. However, because of the emergence of docetaxel-related adverse events (severe allergic reactions), therapy with docetaxel was terminated immediately. Therapy with gemcitabine and oxaliplatin resulted in partial remission followed by splenectomy, retroperitoneal lymph node dissection, segmental resection of the duodenum, and segmental liver resection as well as by a second course of high-dose chemotherapy involving gemcitabine, nab-paclitaxel, and carboplatin with autologous stem cell support. Nearly half a year later, salvage therapy with brentuximab-vedotin was initiated. Afterwards, early staging showed partial remission. Then, after four cycles of brentuximab-vedotin therapy, the addition of pembrolizumab to the treatment regimen led to very good partial remission. Nevertheless, after four cycles of pembrolizumab, this therapy was disrupted due to toxicity (grade 3 immune-mediated hepatitis). Monotherapy with brentuximab-vedotin was continued until new pulmonary lesions appeared, when pembrolizumab was added to brentuximab-vedotin again. Then, the brentuximab-vedotin therapy was stopped because of grade 3 polyneuropathy. Ongoing immunotherapy with pembrolizumab has resulted in regression of all lesions confirmed by CT scans (40).

#### Combinatorial Targeting of Several Immune Checkpoints

One novel therapeutic option also represents the combinatorial targeting of several immune checkpoints, namely, anti-TIGIT (T cell immunoreceptor with Ig and ITIM domains) treatment in combination with anti-PD-1 agents. This approach is supported by the results of the immunohistochemical analysis of 78 seminoma samples performed by Hinsch et al., showing frequent expression of immune checkpoint receptors in these cells. However, it should be noted that high variability in the relative prevalence of TIGIT+ and PD-1+ cells between enrolled patients was detected (49).

Additional promising therapeutic targets in immunotherapy of testicular tumors are T-cell immunoglobulin and mucin domain-3 (TIM-3). Currently published papers have reported that TIM-3 plays a significant role in T-cell exhaustion. In particular, inhibition of the PD-1 pathway may not be sufficient to overcome dysfunction in exhausted T cells, which results in failure of PD-1 monotherapy blockade or adaptive resistance to anti-PD-1 treatment. In this case, the expression of TIM-3 as an alternative immune checkpoint is elevated. Therefore, combined blockade of PD-1 and TIM-3 may represent an effective solution for overcoming resistance to anti-PD-1 therapy (50, 51). Additionally, therapy based on inhibition of immune checkpoint lymphocyte activation gene-3 (LAG-3) should be mentioned in fighting against resistance to anti-PD-1 treatment. LAG-3 is involved in immune homeostasis *via* suppression of T cell activation and cytokine secretion. The remarkable synergistic effect of the combination treatment of anti-LAG-3 and anti-PD-1 has been found in different malignancies, which is also supported by data showing a significant correlation between elevated expression of LAG-3 on TILs and PD-1/PD-L1 expression (52). However, it should be noted that data published by Tu et al. (53) showed no significant difference in LAG-3 and TIM-3 expression between testicular germ cell tumors and/or ovarian cancer and corresponding normal adjacent tissues.

#### Combining of Hypomethylating Agents With Immunotherapy

Data published by Stone et al. provide an interesting background for the combinatorial use of hypomethylating agents and immunotherapy. It is generally known that seminomas are constitutively hypomethylated tumors. The profound hypomethylation of seminoma DNA, correlating also with the abundance of CD8+ cells, was associated with significant expression of human endogenous retroviruses as well as an increase in IFN-α1. Subsequently, activation of type I IFN signaling, as a consequence of the activity of hypomethylating agents, resulted in a more immunogenic phenotype of these tumors (54).

#### Novel Aspect of Patients' Anti-tumor Activity

In addition, Pearce et al. described a tumor-specific immune response mediated by MAGE-specific T cells in patients suffering from GCTs. These data suggest one of the novel aspects of antitumor immunity detected in these patients (16). Another approach may represent TAM-centered (tumor-associated macrophages) anticancer therapy, which is based on inhibition of macrophage recruitment and/or their survival in tumors. Moreover, functional reeducation of TAMs into antitumor agents, manipulation of macrophage-mediated extracellular killing, or phagocytosis as well as intracellular destruction of malignant cells represent key players in this approach (29).

## Conclusion and Future Directions

According to the abovementioned data, we can conclude that the results of a phase II clinical trial evaluating the efficiency of immune checkpoint inhibitors in germ cell tumors are contradictory with previously published case reports and small series (Table 2). A possible explanation for this observation may be the insufficient ability of immune checkpoint inhibitors to eliminate the immune tolerance characteristic of GCTs. According to Jennewein et al., testicular tumors are characterized by a physiologically suppressed immunologic microenvironment. A pathological vascular system in combination with an impaired blood-testis barrier is associated with an increased influx of cytotoxic immune cells into tumor tissue. Nevertheless, due to their exposure to inhibition through PD-L1-mediated immune escape, these cells cannot carry out their antitumor and cytotoxic activities. Subsequently, an anti-angiogenic treatment might be responsible for the reduction of the anti-PD-L1/PD-1 treatment effect. However, anti-angiogenic therapy may also result in normalizing the abnormal structure and function of the tumor vasculature and ultimately contribute to more efficient anti-PD-L1/PD-1 drug delivery (22). Similarly, a low mutation burden described in TGCTs (42), resulting in a low number of neoantigens, could also participate in the deficient clinical activity of these agents. Although the use of immune checkpoint inhibitors (involving PD-L1 and CTLA-4 inhibitors) failed to produce clinically meaningful responses in cisplatin-refractory TGCTs, the possible success of different immune therapy checkpoint inhibitors (other than PD-L1 + CTLA-4 inhibitors) might result in better clinical outcomes. However, to date immune checkpoint inhibition has not been shown to play a role in refractory germ cell tumor treatment outside clinical trials. While a case reports suggested that there exists a population of patients who could potentially benefit from immune checkpoint inhibition, we currently lack valid predictors for clinical decision-making in routine practice. Data from GTS suggest that high expression of PD-L1 as seen in choriocarcinoma could potentially be a relevant biomarker; at present, however, data for such decision-making in TGCTs are lacking. TILs and/or the tumor mutation burden could be potentially alternative predictors; however, further research is needed to determine their utility. At the same time, the clinical benefits of the combination of immunotherapy with conventional cisplatin-based chemotherapy and/or their use in earlier lines of treatment cannot be ruled out. Therefore, further scientific studies are needed to shed light on the determination of prognostic factors predicting the response to immune-based therapy in refractory testicular cancer patients.

## Author Contributions

KK and MM participated in the conception of this review. KK drafted the manuscript. KK, SS, MC, and MM reviewed it critically for important intellectual content. All authors have read and approved the final version of the manuscript for publication.

## Conflict of Interest

The authors declare that the research was conducted in the absence of any commercial or financial relationships that could be construed as a potential conflict of interest.
